# Feasibility of multimodal intraoperative neurophysiological monitoring for extramedullary spinal cord tumor surgery in elderly patients

**DOI:** 10.1007/s00701-023-05682-8

**Published:** 2023-06-24

**Authors:** Sebastian Siller, Akela Sixta, Joerg-Christian Tonn, Andrea Szelenyi

**Affiliations:** Department of Neurosurgery, University Hospital, LMU Munich, Marchioninistrasse 15, 81377 Munich, Germany

**Keywords:** Extramedullary spinal cord tumor surgery, EMSCT, Intraoperative neurophysiological monitoring, IONM, Age, Elderly

## Abstract

**Background/purpose:**

Extramedullary spinal cord tumors (EMSCTs) are mostly benign tumors which are increasingly diagnosed and operatively treated in the elderly. While there are hints that multimodal intraoperative neurophysiological monitoring (IONM) could be influenced by age and age-related comorbidities, no study has ever systematically evaluated its feasibility and value for EMSCT surgery in elderly patients.

**Methods:**

We retrospectively evaluated all patients with microsurgical EMSCT resection under continuous multimodal IONM with SSEPs, MEPs and electromyography between 2016 and 2020. Epidemiological, clinical, imaging and operative/IONM records as well as detailed individual outcomes were analyzed and compared for the cohort < / ≥ 65 years.

**Results:**

Mean age was 45 years in cohort < 65 years (*n* = 109) and 76 years in cohort ≥ 65 years (*n* = 64), while baseline/operative characteristics did not significantly differ. Mean baseline SSEPs’ latencies (left–right average) were significantly higher in the cohort ≥ 65 years for both median (20.9 ms vs. 22.1 ms; *p* < 0.01) and tibial nerve (42.9 ms vs. 46.1 ms; *p* < 0.01) without significant differences for SSEPs’ amplitudes. Stimulation intensity to elicit intraoperative MEPs was significantly higher in the cohort ≥ 65 years (surrogate-marker: left–right-averaged quotient ID1-muscle/abductor-hallucis-muscle; 1.6 vs. 2.1; *p* < 0.001). Intraoperatively, SSEP and MEP monitoring were feasible in 99%/100% and 99%/98% for the cohort < / ≥ 65 years without significant differences in rates for significant IONM changes during surgery or postoperatively new sensorimotor deficits. Sensitivity of IONM was 29%/43%, specificity 99%/98%, positive and negative predictive values 67%/75% and 95%/93% for the cohort < / ≥ 65 years. Overall, age was no risk factor for IONM feasibility or rate of significant IONM changes.

**Discussion:**

Multimodal IONM is feasible/reliable for EMSCT surgery in elderly patients. An age-related prolongation of SSEPs’ latencies and demand for higher stimulation intensities for MEPs’ elicitation has to be considered.

## Introduction

Today, microsurgical resection with intraoperative neurophysiological monitoring (IONM) is widely considered to be the gold standard for intramedullary spinal cord tumor (IMSCT) surgery [24, 25]. Even still under debate, there are also hints in literature that multimodal IONM with motor and somatosensory evoked potentials (MEPs and SSEPs) as well as free-running electromyography (frEMG) can also improve clinical outcome and minimize the risk of iatrogenic injury to neural structures during extramedullary spinal cord tumor (EMSCT) surgery [2, 7, 14, 16, 17, 28].

EMSCTs are mostly benign tumors and meningiomas comprise the vast majority (> 50%), followed by nerve sheath tumors like schwannomas or neurofibromas (ca. 30%) [22]. The peak incidence of these EMSCT modalities has been reported to be in the 5th until 7th decade in the past [22]; however, due to the demographic change with an aging population in the western countries, up-to-date peak incidences of, e.g. benign spinal meningiomas are reported to be in the age of 65–84 years [15].

In turn, evoked potentials can be notably influenced by age with hints for prolonged latencies of both SSEPs [1] and MEPs [4] as well as reduced MEPs’ amplitudes [12, 27], even in the physiological status and especially in the lower limbs. Moreover, the elderly population is prone to higher rates of comorbidities, like diabetes, hypertension, peripheral neuropathy and cervical spondylotic myelopathy, which are known risk factors for a lower success rate of obtaining reliable evoked potentials intraoperatively [10, 26]. There are hints in literature that lower limbs’ MEP monitoring during spinal cord tumor surgery could be less successful in elderly patients over 64 years [6, 23]. Such, higher comorbidity and a potentially higher failure rate for IONM might result in lower IONM success-rate and higher surgical morbidity.

However, up to date, no study has so far specifically addressed the feasibility of multimodal IONM for EMSCT surgery in elderly patients and its predictive value for the neurological and functional outcome. Therefore, in our retrospective study here, we aim to elucidate these aspects by making a comparison with a younger reference group and particularly addressing the following three key points:

i) Are there age-related differences in the feasibility of multimodal IONM for EMSCT surgery and are there specific technical and practical issues to be considered with regard to ensuring a high rate of IONM monitorability in the higher age?
ii) Are there age-related differences in the occurrence rate of critical IONM changes during surgery or differences in the predictive value of IONM for EMSCT surgery?
iii) Are there any risk factors for a poorer IONM feasibility and a higher rate of intraoperative changes in EMSCT surgery?

## Methods and materials

We included all patients who had been referred to our institute (Neurosurgical Clinic, University of Munich, LMU, Germany) from January 2016 to October 2020 for multimodal IONM-aided microsurgical resection of an EMSCT. After study approval by the local Institutional Review Board (AZ20-397), data of these patients were retrospectively enrolled. We collected and analyzed epidemiological and baseline characteristics as well as imaging findings and surgical findings in all patients. According to the three key aims of our study, special emphasis was placed on IONM findings as well as their correlation with neurological and functional outcome measurements as described in the following:

### Surgical procedures and intraoperative neurophysiological monitoring (IONM) setup

Evaluation of tumor location, spinal level and distribution within the spinal cord based on preoperative contrast-enhanced MR imaging. On the basis of radiographical and intraoperative observations, tumors were characterized in terms of their position to the spinal cord as either dorsal or ventral and as either left- or right-sided; all tumors were located completely extramedullary.

Anesthesia for surgery was performed with total intravenous anesthesia, carefully avoiding the application of muscle relaxants despite for intubation purposes. All patients were administered steroids preoperatively. For tumor resection, patients were placed in prone position. Via a posterior midline approach, the lamina and spinous processes overlying the tumor were exposed and either laminotomy, hemilaminectomy, laminoplasty or interlaminar fenestration was performed to provide exposure of the tumor margins. In all cases, intraoperative ultrasonography was used before dural opening to assure a precise exposure of the tumor.

Once the tumor was identified, resection was performed under microscope and ultrasound guidance according to the state-of the-art microsurgical techniques as well as with continuous multimodal IONM as described below. After termination of resection, the dura was closed in a watertight manner and a subdural hematoma was excluded by a subsequent intraoperative ultrasonographic control before tissue and wound closure. All histological samples were reviewed by an experienced neuropathologist and classified based on the 2016 WHO classification of CNS tumors or head/neck tumors or respective classification systems [13, 18]. The final diagnoses reported here are based on the combination of histology, and (where available) genetics and methylome classifier results. Gross-total resection was defined as complete tumor removal according to intraoperative microscopic findings and postoperative contrast-enhanced T1-weighted MR imaging.

In all cases, microsurgical tumor resection was performed under continuous IONM of both SSEPs and transcranial muscle MEPs (TcMEPs) as well as frEMG and direct spinal root stimulation. For stimulating and recording, an integrated IONM system was used (ISIS, Inomed Co., Germany). To elicit TcMEP recording, stimulating electrodes placed at C1, C3, C2, C4 (acc. to the international 10–20-EEG-system) and TcMEP were elicited in a medial or lateral interhemispheric fashion. A constant current stimulator (250 mA at 400 V maximum) delivered anodal train-of five pulses (250–500 Hz, 0.5 ms individual pulse width) at a 0.5–1 Hz repetition rate. For recording, subdermal pairs of electrodes were placed bilaterally and in thenar, hypothenar, M. interosseus digiti 1, M. pollicis brevis, M. abductor minimus and tibialis anterior muscles, as well as additionally in the biceps, brachioradialis, triceps, iliopsoas, adductor, quadriceps femoris, tibialis anterior, foot flexor, extensor hallucis longus, abductor hallucis and/or sphincter muscle groups according to the spinal level of interest. SSEPs (individual pulse width 200–500 ms, 2.7–4.7 Hz repetition rate, maximum 40 mA, at least 200 averages) were performed in median and posterior tibial nerve as well as and pudendal nerve according to the spinal level of interest.

After patient positioning, utmost care was taken to optimize and adjust stimulation and recording parameters to obtain SEP and TcMEP responses. Baseline parameters (incl. stimulation intensity, amplitude and latency) were then documented and compared between the patients’ cohort < resp. ≥ 65 years of age (cut-off was selected according to hints of age-related differences in IONM in literature [1, 4, 6, 10, 12, 26, 27]). The rate of successful obtainment of baseline signals for each IONM modality (and for each upper and lower limb) was also analyzed and compared between the cohort < resp. ≥ 65 years of age.

### Critical IONM changes during surgery and predictive value of IONM

SSEPs and TcMEPs were continuously recorded in an alternating fashion throughout surgery; if TcMEP-related patient movement was considered disturbing during tumor microdissection, TcMEPs were performed frequently, but only on surgeons’ request (especially after manipulation of the tumor or the spinal cord) and while pausing dissection. To identify cervical and lumbosacral motor nerve roots, direct stimulation (with a handheld bipolar concentric probe) was used to elicit compound muscle action potentials (CMAPs). Electrophysiological data were continuously analyzed by a technician trained in IONM and supervised by an experienced senior neurophysiologist. Any IONM changes were immediately issued to the surgeons’ team and the interpretation was performed interdisciplinary between the surgical and electrophysiological team.

Significant reduction in SSEP amplitude ≥ 50% and/or an increase in SSEP latency ≥ 2 ms as well as significant decrement in TcMEPs amplitude ≥ 80% as well as significant spontaneous EMG activity (e.g., neurotonic discharges) especially during or immediately after surgical manipulation were defined as ‘warning criteria of IONM’ and—if technical reasons (e.g. dislocation of electrodes), anesthesiological reasons (e.g. lowering of blood pressure or body temperature, change of intravenous anesthesia management or addition of volatile anesthetics) and temporary surgical reasons (e.g. irrigation with cold saline solution) are excluded—classified as ‘critical IONM changes’.

In circumstances of critical IONM changes, first technical problems were ruled out and then the most recent anesthesiological and surgical steps were reconsidered, and immediate corrective actions were initiated, e.g. cessation of additional volatile anesthetics, correction of blood pressure or modification of the surgical technique (e.g. temporary haltering of resection, reduction of traction on the tumor or surrounding tissue, irrigation with warm saline solution and/or continuation of resection at distant sites). In case of repetitive (or persistent) critical IONM changes during the resumption of tumor resection/dissection in the very same area where prior manipulation has been halted for corrective actions due to critical IONM changes, further attempts for additional tumor resection/dissection were abandoned. For statistical analysis, critical IONM changes in SSEPs, TcMEPs and/or EMG monitoring were classified as either ‘transient pathological’ (complete resolution within surgery till dura closure) or ‘permanent pathological’ (persistence after dura closure) for the respective IONM modality, while no critical IONM changes were classified as ‘non-pathological IONM’.

For correlation analyses, the following outcome parameters were assessed: detailed neurological status (including sensory, reflex, muscle tonus and gait examinations) and fine-motor evaluation via the Medical Research Council (MRC) grading system for muscle strength [8] were performed in all patients before and after surgery as well as the most recent follow-up timepoint afterwards (termed as ‘last follow-up’ in the following); postoperatively new or worsened sensorimotor deficits and bladder/bowel dysfunction as well as pain or painful dysaesthesia were also recorded. The classification of McCormick [20] and the modified Japanese Orthopaedic Association Score (mJOA) [3] were used for functional evaluation, while the Visual Analogue Scale for pain and Barthel Index (BI) [19] were used for the assessment of pain-related disability and general performance in daily life before and after surgery as well as at last follow-up visit. The Odom score [21] and Patient Satisfaction Index (PSI) [9] were used to evaluate the general postoperative and follow-up outcome and subjective satisfaction.

To investigate the predictive value of multimodal IONM for the detailed upper and lower limbs’ (fine-motor) function during the postoperative course, intraoperative findings (‘permanent pathological’) of the aforementioned IONM modalities (and any combination of them) were correlated with the limbs’ motor/neurological outcome measurements as well as abovementioned outcome compound scores preoperatively and at the abovementioned postoperative timepoints and compared with regard to sensitivity, specificity, positive/negative predictive values (PPV/NPV) for the patients’ cohort < resp. ≥ 65 years of age.

### Risk factors and statistical analysis

Statistical analysis was performed using Sigma Plot for Windows v.11 (Systat Software Inc., USA). Differences were defined to be statistically significant if the *p*-value is < 0.05. For comparison of groups for differences, the Student’s *t*-test was used for numeric values, the Mann–Whitney Rank Sum test for ordinal variables and the *χ*^2^-test resp. Fisher’s exact test (in case of 2 × 2-contingency tables) for nominal variables. To investigate risk factors (including patient sex/age/body-mass-index [BMI], kind/duration of clinical symptoms and tumor location related to the myelon, tumor extent and entity, etc.) associated with (1) successful obtaining of IONM baseline signals and IONM monitorability during the intraoperative course as well as (2) occurrence of transient/permanent critical IONM changes during tumor resection, logistic regression analyses (polytomous variables) and *χ*^2^-test resp. Fisher’s exact tests (dichotomous variables) were performed. This article adheres to the Strengthening the Reporting of Observational Studies in Epidemiology guidelines for reporting [30].

## Results

### Patient and tumor characteristics

Altogether, 173 patients with EMSCTs were operated in our neurosurgical university center during the 5-year observational period. There was a predominance of the female gender (male/female: 1:1.5) and arterial hypertension was the most frequent comorbidity (*n* = 52, 30.1%). Pain (*n* = 119, 68.8%) and sensory deficits (*n* = 114, 65.9%) were the most frequent preoperative complaints with a mean duration of symptoms of 15.8 months. Tumors were predominantly located in the thoracic spine (*n* = 80, 46.2%) and of two-level extent (*n* = 111, 64.2%) with all tumors being benign (WHO grade 1) as well as strictly intradural and completely extramedullary; meningiomas (*n* = 83, 48.0%) and schwannomas (*n* = 80, 46.2%) were the most frequent tumor entities.

At the timepoint of surgery, 109 patients were < 65 years of age and 64 patients ≥ 65 years. Baseline patients’ and tumor characteristics for each cohort are displayed in Table 1 and did not significantly differ between both cohorts.

**Table 1:** Baseline characteristics at admission for surgery^*^

Characteristics	Patients		*p*-value
	< 65 years (*n* = 109)	≥ 65 years (*n* = 64)	
Gender, male/female	40/69	17/47	0.880
Median age, years (range)	47 (18–64)	75 (66–90)	< 0.001
Comorbidities, no. (%)			
Cardiovascular diseases	1 (0.9)	4 (6.3)	1.000
Arterial hypertension	16 (14.7)	36 (56.3)	0.488
Diabetes mellitus	3 (2.8)	8 (12.5)	0.799
Polyneuropathy	1 (0.9)	10 (15.6)	0.156
BMI, kg/m^2^	24.8 ± 4.5	26.1 ± 4.5	0.082
Preoperative symptoms, no. (%)			
Motor deficit	34 (31.2)	24 (37.5)	0.751
Sensory deficit	76 (69.7)	38 (59.4)	0.903
Gait ataxia	27 (24.8)	36 (56.3)	0.322
Vegetative symptoms	10 (9.2)	6 (9.4)	1.000
Pain	79 (72.5)	40 (62.5)	1.000
Mean duration of symptoms, months	12.2 ± 15.6	15.8 ± 21.6	0.256
Tumor entity, no. (%)			
Meningeoma	33 (30.3)	50 (78.1)	0.050
Schwannoma	66 (60.6)	14 (21.9)	
Other	10 (9.1)	0 (0.0)	
Location of tumor, no. (%)			
Cervical	34 (31.2)	19 (29.7)	0.089
Thoracic	42 (38.5)	38 (59.4)	
Lumbar	33 (30.3)	7 (10.9)	
Extent of tumor, no. (%)			
Singel-level	35 (32.1)	17 (26.6)	0.610
Two-level	67 (61.5)	44 (68.8)	
Three-level or more	7 (6.4)	3 (4.6)	

^*^Mean values are presented ± standard deviation

### Surgical characteristics and feasibility of IONM

There were no significant differences in surgical characteristics between the cohort < resp. ≥ 65 years: Sufficient exposure of the tumor for resection was achieved via laminotomy in 32.8% resp. 10.9% of the cases, hemilaminectomy in 48.6% resp. 57.8%, laminectomy in 13.8% resp. 31.3% and laminoplasty in 2.8% resp. 0% (*p* = 0.146). Mean operation time (from skin incision till skin closure) was 200 ± 76 min resp. 204 ± 58 min (*p* = 0.654) and mean intraoperative blood loss was 292 ± 306 ml resp. 252 ± 194 ml (*p* = 0.346); gross-total tumor resection according to intraoperative findings and postoperative/follow-up MRI findings was accomplished in 87.2% and 90.6% (*p* = 0.095).

Intraoperatively, EMG monitoring of limbs’ and anal sphincter muscles as well as direct nerve stimulation (whenever applied) was feasible in all cases of both cohorts. With a 45-year-old male patient with a large schwannoma in the L5/S1-level lacking to obtain lower limbs’ MEPs and a 37-year-old male patient with neurofibromatosis and a large neurofibroma in the L4/5-level lacking to obtain both median and tibial nerve SSEPs, obtainment of baseline signals for unimpaired intraoperative monitoring was feasible in 99.1% of the cases both for SSEPs and MEPs in the cohort < 65 years. In the cohort ≥ 65 years, MEPs of the lower limbs’ muscles could not be obtained (from the timepoint of baseline acquisition on) in an 83-year-old female patient with a meningioma in the levels Th2-Th4, while baseline SSEPs could be recorded in all cases of this cohort; feasibility of MEP and SSEP monitoring was therefore 98.4% and 100% in the cohort ≥ 65 years. There were no significant differences in rates of IONM feasibility between both cohorts (*p*_MEPs_ = 1.000, *p*_SSEPs_ = 1.000).

Mean baseline left–right average SSEPs’ latencies were significantly higher in the cohort ≥ 65 years for both median nerve (20.8 ± 3.0 ms vs. 22.1 ± 2.1 ms; *p* = 0.006) and tibial nerve (42.9 ± 5.4 ms vs. 46.1 ± 4.8 ms; *p* < 0.001), while mean baseline left–right average SSEPs’ amplitudes for median nerve (3.2 ± 2.1 mV vs. 4.0 ± 2.9 mV; *p* = 0.053) and tibial nerve (2.2 ± 3.4 mV vs. 1.6 ± 1.8 mV; *p* = 0.203) did not significantly differ between both cohorts. To compare the stimulation intensity needed to elicit MEPs, the left–right average stimulation intensity for the M. interosseus D1 (surrogate marker for the upper limbs) and M. abductor hallucis (surrogate marker for the lower limbs) was determined: while left–right average stimulation intensity for the obtainment of M. interosseus D1 MEPs was similar in both cohorts (54.0 ± 17.7 mA vs. 52.5 ± 23.8 mA; *p* = 0.689), stimulation intensity to elicit intraoperative M. abductor hallucis MEPs was significantly higher in the cohort ≥ 65 years (83.8 ± 22.0 mA vs. 103.6 ± 36.0 mA; *p* < 0.001). Correspondingly, the left–right-averaged stimulation intensity quotient M. interosseus D1 / M. abductor hallucis MEPs was 1.6 vs. 2.1 (*p* < 0.001), reflecting the demand for significantly higher stimulation intensities to obtain MEPs in the lower extremities in the cohort ≥ 65 years.

### Critical IONM changes during surgery and predictive value of IONM

In all patients, anesthesiological and surgical corrective actions were always stepwise initiated as described in the ‘Methods and materials’ section whenever critical IONM changes above the ‘warning criteria’ occurred during surgical approach or tumor resection and technical reasons could be excluded.

This was the case in three patients (2.8%) of the cohort < 65 years: (i) a 48-year-old female patient with a calcified anterior-lateral intradural-extramedullary meningioma in the levels Th5-Th6 with permanent loss of lower limbs’ MEPs on the left side during laminectomy and transient loss of lower limbs’ MEPs on the right side during tumor resection (after dura closure, lower limbs’ MEPs on the right side were recorded with small amplitudes under markedly increased stimulation intensity) followed by a postoperative new left-dominated paraparesis with only incomplete recovery during long-term follow-up; (ii) an 18-year-old male patient with C5/6-schwannoma with significant, but transient amplitude reductions of the right upper and lower limbs during dissection of the tumor masses without postoperative changes in motor function; and (iii) an 18-year-old female neurofibromatosis patient with a C4-C7 neurofibroma with transient, but significant amplitude reduction of the left upper limbs’ MEPs and permanent significant median nerve SSEP latency prolongation during tumor resection (which was abandoned at this timepoint) follow by a transient postoperative left-sided upper limbs’ paresis and a permanent sensory deficit of the left hand.

In the cohort ≥ 65 years, this was the case in four patients (6.3%): (i) an 80-year-old female patient with a lateral meningioma in the Th10/11-level with a loss of left-sided tibialis anterior and foot flexor MEPs during tumor resection (which was abandoned at this timepoint) followed by a postoperative transient new paresis of the left lower limb; (ii) a 75-year-old female patient with a dorsolateral Th4/5-meningioma with transient loss of the lower limbs’ MEPs on the left side during the late stage of hemilaminectomy procedure and initial stage of tumor dissection followed with a transient paresis of the left lower limb; (iii) a 73-year-old female patient with a lateral meningioma in the Th9/10-level with a significant increase in MEPs’ stimulation intensity for the lower limbs and a transient loss of the quadriceps femoris and tibialis anterior MEPs on the left side during tumor resection which was not accompanied by any changes in neurological status postoperatively; and (iv) an 80-year-old female patient with a lateral meningioma in the Th4/5-level with transient loss of right-sided lower limbs’ MEPs during hemilaminectomy procedure and a 60% permanent amplitude reduction of the left tibial nerve SSEP (occurring during tumor resection) with a transient postoperative right-dominated paraparesis and hypesthesia of the left lower limbs. Statistical analysis did not reveal any significant differences in the occurrence of transient/permanent critical IONM changes during surgical approach and tumor resection between the cohort < resp. ≥ 65 years (*p*_IONM_ = 0.126; *p*_MEPs_ = 1.000; *p*_SSEPs_ = 1.000).

With one 57-year-old male patient with a C5/6-schwannoma and one 58-year-old female patient with a L1/2-schwannoma suffering from a postoperatively new transient paresis as well as one 58-year-old female patient with a C3/4-schwannoma suffering from a transient and two patients (a 27-year-old female patient with a C6/7-schwannoma and a 50-year-old male patient with a L2/3-schwannoma) suffering from a permanent postoperatively new sensory deficit of both lower limbs despite completely unremarkable intraoperative IONM course, the sensitivity of multimodal IONM to predict postoperative neurological deficits (matching the IONM modality) was 28.6%, specificity 99.0%, positive and negative predictive values (PPV and NPV) 66.7% and 95.3% for the cohort < 65 years.

With two female patients (69- and 84-year-old) with a mid-thoracic meningioma suffering from a postoperatively new transient paresis as well as one 74-year-old female patient with a C3/4-meningioma suffering from a transient and one 72-year-old male patient with a C5/6-meningioma suffering from a permanent postoperative new sensory deficit despite completely unremarkable intraoperative IONM course, the corresponding sensitivity was 42.9%, specificity 98.2%, PPV 75.0% and NPV 93.3% for the cohort ≥ 65 years. A comprehensive overview of IONM characteristics in the cohort < and ≥ 65 years is given in Table 2.

**Table 2:** IONM characteristics^*^

Characteristics	Patients		*p*-value
	< 65 years (*n* = 109)	≥ 65 years (*n* = 64)	
IONM monitorability, no. (%)			
TcMEPs	108 (99.1)	63 (98.4)	1.000
SSEPs	108 (99.1)	64 (100.0)	1.000
EMG/direct nerve stimulation	109 (100.0)	64 (100.0)	1.000
Mean baseline left–right average stimulation intensity, mA			
M. interosseus D1	54.0 ± 17.7	52.5 ± 23.8	0.689
M. abductor hallucis	83.8 ± 22.0	103.6 ± 36.0	< 0.001
Left–right-averaged stimulation intensity quotient M. interosseus D1/M. abductor hallucis MEPs	1.6	2.1	< 0.001
Mean baseline left–right average SSEPs’ latencies, ms			
Median nerve	20.8 ± 3.0	22.1 ± 2.1	0.006
Tibial nerve	42.9 ± 5.4	46.1 ± 4.8	< 0.001
Mean baseline left–right average SSEPs’ amplitudes, mV			
Median nerve	3.2 ± 2.1	4.0 ± 2.9	0.053
Tibial nerve	2.2 ± 3.4	1.6 ± 1.8	0.203
Rate of critical IONM changes, no. (%)	3 (2.8)	4 (6.3)	0.126
IONM accuracy, no. (%)			
True-positive cases	2 (1.8)	3 (4.7)	1.000
False-positive cases	1 (0.9)	1 (1.6)	
True-negative cases	101 (92.7)	56 (87.5)	
False-negative cases	5 (4.6)	4 (6.3)	
IONM performance, %			
Sensitivity	28.6	42.9	1.000
Specificity	99.0	98.2	
Positive predictive value	66.7	75.0	
Negative predictive value	95.3	93.3	

^*^Mean values are presented ± standard deviation

All in all, the rates for new neurological deficits postoperatively were as follows: 3.7% of the patients had a transient new motor deficit (permanent in 0.9%) and 4.6% a new sensory deficit (permanent in 3.7%) for the cohort < 65 years, while 6.3% had a transient new motor deficit and 4.7% a new sensory deficit (permanent in 1.6%) in the cohort ≥ 65 years (no significant differences between both cohorts; *p* = 1.000). The duration of transient new motor and sensory deficits did also not significantly differ between the cohort < and ≥ 65 years (*p* = 1.000).

Postoperative functional status outcome at discharge and last follow-up (compared to the timepoint of admission) separated for the cohort < resp. ≥ 65 years are displayed in Table 3. In both cohorts, pain level (*p* < 0.001 vs. *p* < 0.001) and myelopathic symptomatology (mJOA score and McCormick score as surrogate parameters) were significantly improved at the timepoint of last follow-up (*p* < 0.001 and *p* < 0.001 vs. *p* = 0.026 and *p* = 0.014). Mean length of in-patients stay was significantly longer in patients of the cohort ≥ 65 years (10.4 days vs. 8.2. days; *p* < 0.001). However, extent of postoperative and long-term improvement of pain situation (VAS), myelopathic symptomatology (mJAO and McComick score) and general performance in daily-life (BI) were similar between both cohorts without any significant differences; there was also no difference (each *p* = 1.000) in both cohorts for the rate of surgical complications and rate of reoperation due to distant spinal tumor remanifestation during long-term follow-up, proving EMSCT resection with multimodal IONM to be safe and effective even in elderly patients.

**Table 3:** Functional status and postoperative outcome^*^

Characteristics	Patients		*p*-value
	< 65 years	≥ 65 years	
VAS, median (range)			
Preoperative	3 (0–9)	4 (0–9)	0.607
At discharge after reoperation	2 (0–6)	2 (0–7)	0.626
At last follow-up	0 (0–6)	0 (0–4)	0.870
Difference preoperative vs. at discharge, *p*-value	0.002	0.005	0.916
Difference preoperative vs. at last follow-up, *p*-value	< 0.001	< 0.001	0.399
mJOA, median (range)			
Preoperative	18 (8–18)	16 (11–18)	< 0.001
At discharge after reoperation	18 (9–18)	16 (12–18)	< 0.001
At last follow-up	18 (14–18)	17 (14–18)	< 0.001
Difference preoperative vs. at discharge, *p*-value	0.485	0.776	0.821
Difference preoperative vs. at last follow-up, *p*-value	< 0.001	0.026	0.892
McCormick score, median (range)			
Preoperative	1 (1–4)	2 (1–4)	< 0.001
At discharge after reoperation	2 (1–4)	2 (1–4)	< 0.001
At last follow-up	1 (1–3)	1.5 (1–3)	< 0.001
Difference preoperative vs. at discharge, *p*-value	0.007	0.414	0.555
Difference preoperative vs. at last follow-up, *p*-value	< 0.001	0.014	0.932
Barthel Index, median (range)			
Preoperative	100 (40–100)	100 (45–100)	0.122
At discharge after reoperation	100 (50–100)	100 (45–100)	0.195
At last follow-up	100 (75–100)	100 (90–100)	0.151
Difference preoperative vs. at discharge, *p*-value	0.550	0.804	0.838
Difference preoperative vs. at last follow-up, *p*-value	0.218	0.325	0.979
PSI, median (range)			
At last follow-up	1 (1–4)	1 (1–4)	0.296
Odom score, median (range)			
At last follow-up	2 (1–4)	2 (1–4)	0.298
Mean length of inpatient-stay, days ± SD	8.2 ± 2.7	10.4 ± 3.9	< 0.001
Rate of reoperation due to distant spinal tumor remanifestation, no. (%)	4 (3.7)	1 (1.6)	1.000
Mean follow-up, months ± SD	14.8 ± 15.2	17.2 ± 17.9	0.423

^*^Mean values are presented ± standard deviation

### Risk factors for IONM feasibility and intraoperative changes

Prognostic modelling for successfully obtaining IONM baseline signals and IONM monitorability during the intraoperative course as well as for occurrence of transient/permanent critical IONM changes during tumor resection are summarized in Table 4. Overall, age was not identified to be a negative prognostic risk factor for either IONM feasibility or rate of critical IONM changes during EMSCT surgery.

**Table 4:** Prognostic factors

Variables	IONM feasibility OR (*p*-value/95%-CI)	critical IONM changes OR (*p*-value/95%-CI)
Gender		
Male vs. female	0.15 (0.341/0.01–7.73)	0.46 (0.515/0.904–4.82)
Age (years)		
Per year	0.98 (0.715/0.85–1.12)	0.92 (0.067/0.83–1.01)
Cardiovascular diseases		
Yes vs. no	< 0.01 (0.999/0.00–inf)	< 0.01 (0.999/0.00–inf)
Arterial hypertension		
Yes vs. no	1.56 (0.856/0.02–193.0)	3.01 (0.421/0.21–43.9)
Diabetes mellitus		
Yes vs. no	< 0.01 (0.999/0.00–inf)	1.27 (0.753/0.29–5.58)
Polyneuropathy		
Yes vs. no	< 0.01 (0.999/0.00–inf)	7.51 (0.323/0.14–409.5)
BMI (kg/m^2^)		
Per kg/m^2^	0.61 (0.063/0.36–1.03)	1.06 (0.642/0.84–1.34)
Preoperative motor deficit		
Yes vs. no	1.28 (0.935/ < 0.01–486.4)	1.66 (0.644/0.19–14.2)
preoperative sensory deficit		
Yes vs. no	0.03 (0.241/ < 0.01–10.9)	1.07 (0.959/ < 0.01–14.1)
Preoperative gait ataxia		
Yes vs. no	17.6 (0.267/0.11–2774.3)	0.82 (0.884/0.05–12.4)
Preoperative vegetative symptoms		
Yes vs. no	3.5 (0.144/0.65–19.1)	< 0.01 (0.997/0.00–inf.)
Preoperative mJOA score		
mJOA score	0.88 (0.586/0.56–1.38)	0.99 (0.982/0.67–1.48)
Preoperative Barthel Index score		
Barthel Index score	0.98 (0.452/0.92–1.04)	1.04 (0.667/0.90–1.18)
Tumor entity		
Meningioma vs. schwannoma vs. other	0.29 (0.513/0.01–11.8)	0.18 (0.201/0.01–2.47)
Tumor location		
Spinal level	1.16 (0.181/0.94–1.43)	0.99 (0.806/0.90–1.09)
Tumor extent		
No. of levels of extent	2.51 (0.441/0.24–26.2)	1.45 (0.731/0.18–12.0)

*OR*, odds ratio; *95%-CI*, 95% confidence interval

## Discussion

Even still under debate, there are increasing hints in literature that multimodal IONM with MEPs and SSEPs as well as frEMG can improve clinical outcome and minimize the risk of iatrogenic injury to neural structures during EMSCT surgery [2, 7, 14, 16, 17, 28]. Today, in the vast majority of EMSCTs, like benign meningiomas and nerve sheath tumors, the peak incidence has shifted from the 5th till 7th decade of life in former times to the 7th till 8th decade now, which has to be seen in the context of demographic changes with a generally aging population in the western countries [15]. Especially in this group of elderly patients, we deem the use of IONM in EMSCT surgery to be particularly important to ensure a maximum safe resection and minimize the risk of a postoperatively deteriorated neurological/functional outcome which would be particularly tremendous in this group of patients with an already age-related higher burden of comorbidities and higher risk of impaired functioning in daily life compared to a younger cohort. Despite it is known from basic research that evoked potentials can be notably influenced by age and age-related comorbidities with possibly lower success rates of obtaining reliable IONM signals during spinal cord tumor surgery in patients ≥ 65 years [1, 4, 6, 10, 12, 23, 26, 27], no study so far has specifically addressed the practical feasibility of multimodal IONM for EMSCT surgery in elderly patients and its predictive value for the detailed neurological outcome. In our retrospective study, we could show that multimodal IONM was feasible for EMSCT surgery in elderly patients. However, an age-related upfront prolongation of SSEPs’ latencies and a demand for higher stimulation intensities for MEPs’ elicitation at baseline have to be considered in elderly patients. No hints for an age-related higher ‘vulnerability’ of the spinal cord during surgery in terms of intraoperative occurrence of critical IONM changes or postoperatively new neurological deficits/functional outcome were found.

### Validity of data and age-related feasibility and practical issues of IONM

In our retrospective study, baseline characteristics of our total study population with a median age in the 5th decade of life, a predominance of the female gender as well as pain and sensory deficits as the most frequent preoperative complaints, were similar to literature data for large EMSCT case series [7, 16, 28]. The same was true for a predominant tumor location in the thoracic spine, for the distribution and grading of tumor entities with WHO grade I meningiomas and nerve sheath tumors as most frequent findings as well as for the surgical characteristic with regard to mean blood loss, mean operation times, rate of complete tumor resection of about 90% and rate of postoperatively new neurological deficits of < 5% which underlines the validity and comparability of our data [7, 16, 17, 28, 29].

While literature data report about a monitorability rate as low as 68% for MEPs and 67% for SSEPs in EMSCT surgery [17, 23], we could show a very high feasibility of > 98% for both MEPs and SSEPs. Within the setting of a very experienced IONM team routinely performing about 500 IONM cases per year, we also could not find any differences between the monitorability of the upper and lower limbs as well as between the cohort < 65 years and ≥ 65 years for both MEPs and SSEPs. Furthermore, regression analysis did not identify age to be significantly associated with the rate of IONM feasibility and monitorability in our study population. This stands in contrast to the publications of Chen et al. [5, 6] that could identify age to be a risk factor for an impaired monitorability rate of at least lower limbs’ MEPs and SSEPs during spine surgery in general, although these two publications only comprised a small portion of patients with EMSCTs and are therefore not reasonably comparable to our study. The only publication so far investigating factors predicting the feasibility of monitoring lower limbs’ MEPs in patients undergoing spinal cord tumor surgery of Rajshekhar et al. [23] also could not identify age to be a risk factor; however, poor functional status and severe muscle weakness prior to surgery were found to be negative predictors. But, the overall low sample size and the mixture of intra- and extramedullary spinal cord tumors limit the validity of the case series of Rajshekhar et al. [23] and intrude the comparability to our study with a homogeneous and large EMSCT study population.

In terms of multimodal IONM in patients ≥ 65 years, however, we could find a demand for higher stimulation intensities to evoke MEPs which is at least necessary for the lower extremities. This is in line with basic research findings about transcranial magnetic stimulation showing that lower limbs’ MEPs’ amplitudes can be notably reduced by age already in the physiological status [12, 27]. Moreover, we could show that baseline SSEPs’ latencies of both upper and lower limbs were prolonged in the cohort ≥ 65 years which also reflects known findings from basic clinical neurophysiological research publications showing an age-related SSEP latencies’ prolongation even in the physiological status [1] and has always to be considered whenever applying multimodal IONM in elderly patients.

### Age-related vulnerability and predictive value of IONM and risk factor analysis

Baseline patients’, tumor and surgical characteristics were similar between our cohort < and ≥ 65 years underlining the comparability of our two cohorts. Since neither occurrence rate of transient/permanent critical IONM changes during surgery nor rate of new sensorimotor deficits postoperatively was significantly different between both our cohorts, we conclude that higher age of life does not per se predestinates for a higher ‘vulnerability’ of the spinal cord during surgery of EMSCTs. Correspondingly, diagnostic values of multimodal IONM like sensitivity and specificity as well as PPV and NPV were similar between the cohort < and ≥ 65 years in our study. Moreover, with a multimodal IONM’s sensitivity of 42.9%, specificity of 98.2%, PPV of 75.0% and NPV of 93.3% for our cohort ≥ 65 years with regard to a postoperatively new neurological deficit (matching the IONM modality), these diagnostic values of multimodal IONM for EMSCT resection in patients ≥ 65 years are in line with current literature data of larger EMSCT case series [16, 17, 29]. Interestingly, when looking at the 7 cases with critical IONM changes above the ‘warning criteria’ during EMSCt surgery in detail, there was a tendency for a domination of root or anterior/posterior horn addicted events in the patient cohort < 65 years, while there was a tendency for a domination of spinal cord tract addicted events in the patient cohort ≥ 65 years. We want to emphasize that we did not observe any complete bilateral MEP losses below the spinal level of interest in our study population; it is in accordance with IONM data that partial or transient MEP loss and serious MEP amplitude deterioration do not result into severe and long-standing neurological impairments [11].

With regard to prognostic modelling, neither age nor any other of the investigated variables could be identified as negative prognostic risk factors for either IONM feasibility or occurrence rate of critical IONM changes during EMSCT surgery in our study population.

## Conclusion

Our study specifically addressed the actual feasibility of multimodal IONM for EMSCT surgery in elderly patients ≥ 65 years and its prognostic value for the detailed neurological and functional outcome. Overall, age was not a negative confounder for IONM monitorability. Multimodal IONM was both feasible and reliable for EMSCT surgery in elderly patients with no hints for a higher ‘vulnerability’ of the spinal cord during surgery in terms of intraoperative occurrence of transient/permanent critical IONM changes or postoperatively new neurological deficits. However, an age-related upfront prolongation of SSEPs’ latencies and a demand for higher stimulation intensities for MEPs’ elicitation at baseline have to be considered in elderly patients. Altogether, we could show that multimodal IONM can be reliably performed in EMSCT surgery in both young and elderly patients without any differences in neurological and functional long-term outcome.

## Data Availability

The data that support the findings of this study are available from the corresponding author, Sebastian Siller, upon reasonable request.

## Abbreviations and acronyms

BI: Barthel Index
BMI: Body mass index
ca.: Circa
CI: Confidence intervall
CMAP: Compound muscle action potential
EMSCT: Extramedullary spinal cord tumor
frEMG: Free-running electromyography
IMSCT: Intramedullary spinal cord tumor
incl.: Including
IONM: Intraoperative neurophysiological monitoring
KPS: Karnofsky Performance Scale
mJOA: Modified Japanese Orthopaedic Association Score
MRC: Medical Research Council
MRI: Magnetic resonance imaging
NDI: Neck Disability Index
NPV: Negative predictive value
PPV: Positive predictive value
ODI: Oswestry Disability Index
PSI: Patient Satisfaction Index
PSS: Performance Status Scale
resp.: Respectively
SD: Standard deviation
SSEPs: Somatosensory evoked potentials
TcMEPs: Transcranially motor evoked potentials
WHO: World Health Organization

## References

[1] Allison T, Wood CC, Goff WR (1983). Brain stem auditory, pattern-reversal visual, and short-latency somatosensory evoked potentials. Latencies in relation to age, sex, and brain and body size. Electroencephalogr Clin Neurophysiol.

[2] Baig MA, Vastani A, Syrris C, Boardman T, Ghani I, Murphy C, Gebreyohanes A, Vergani F, Mirallave-Pescador A, Lavrador JP, Vasan AK, Grahovachttps G (2022) Intraoperative neurophysiological monitoring for intradural extramedullary spinal tumours. Global Spine Journal. 10.1177/21925682221139822 10.1177/21925682221139822 PMC11289564 36411068

[3] Benzel EC, Lancon J, Kesterson L, Hadden T (1991). Cervical laminectomy and dentate ligament section for cervical spondylotic myelopathy. J Spinal Disord.

[4] Cantone M, Lanza G, Vinciguerra L, Puglisi V, Ricceri R, Fisicaro F, Vagli C, Bella R, Ferri R, Pennisi G, Di Lazzaro V, Pennisi M (2019). Age, height, and sex on motor evoked potentials. Translational data from a large Italian cohort in a clinical environment. Front Hum Neurosci.

[5] Chen JH, Shilian P, Cheongsiatmoy J, Gonzalez AA (2018). Factors associated with inadequate intraoperative baseline lower extremity somatosensory evoked potentials. J Clin Neurophysiol.

[6] Chen X, Sterio D, Ming X, Para DD, Butusova M, Tong T, Beric A (2007). Success rate of motor evoked potentials for intraoperative neurophysiologic monitoring. Effects of age, lesion location, and preoperative neurologic deficits. J Clin Neurophysiol.

[7] Cofano F, Giambra C, Costa P, Zeppa P, Bianconi A, Mammi M, Monticelli M, Di Perna G, Junemann CV, Melcarne A, Massaro F, Ducati A, Tartara F, Zenga F, Garbossa D (2020). Management of extramedullary intradural spinal tumors. The impact of clinical status, intraoperative neurophysiological monitoring and surgical approach on outcomes in a 12-year double-center experience. Front Neurol.

[8] Compston A (2010). Aids to the investigation of peripheral nerve injuries. Medical Research Council. Nerve Injuries Research Committee. His Majesty’s Stationery Office: 1942; pp. 48 (iii) and 74 figures and 7 diagrams; with aids to the examination of the peripheral nervous system. By Michael O'Brien for the Guarantors of Brain. Saunders Elsevier: 2010; pp. 8 64 and 94 Figures. Brain.

[9] Daltroy LH, Cats-Baril WL, Katz JN, Fossel AH, Liang MH (1996). The North American spine society lumbar spine outcome assessment Instrument. Reliability and validity tests. Spine (Phila Pa 1976).

[10] Deiner SG, Kwatra SG, Lin H-M, Weisz DJ (2010). Patient characteristics and anesthetic technique are additive but not synergistic predictors of successful motor evoked potential monitoring. Anesth Analg.

[11] Deletis V, Sala F (2008). Intraoperative neurophysiological monitoring of the spinal cord during spinal cord and spine surgery. A review focus on the corticospinal tracts. Clin Neurophysiol.

[12] Eisen A, Siejka S, Schulzer M, Calne D (1991). Age-dependent decline in motor evoked potential (MEP) amplitude. With a comment on changes in Parkinson's disease. Electroencephalogr Clin Neurophysiol.

[13] El-Naggar AK (2017) WHO classification of head and neck toumours, 4th edition. World Health Organization classification of tumours. 4th edition. International Agency for Research on Cancer, Lyon, France

[14] Ghadirpour R, Nasi D, Iaccarino C, Giraldi D, Sabadini R, Motti L, Sala F, Servadei F (2015) Intraoperative neurophysiological monitoring for intradural extramedullary tumors: why not? Clin Neurol Neurosurg 130:140–149. 10.1016/j.clineuro.2015.01.007 10.1016/j.clineuro.2015.01.007 25618840

[15] Ghaffari-Rafi A, Mehdizadeh R, Ghaffari-Rafi S, Leon-Rojas J (2021). Demographic and socioeconomic disparities of benign and malignant spinal meningiomas in the United States. Neurochirurgie.

[16] Ishida W, Casaos J, Chandra A, D'Sa A, Ramhmdani S, Perdomo-Pantoja A, Theodore N, Jallo G, Gokaslan ZL, Wolinsky J-P, Sciubba DM, Bydon A, Witham TF, Lo S-FL (2019) Diagnostic and therapeutic values of intraoperative electrophysiological neuromonitoring during resection of intradural extramedullary spinal tumors. A single-center retrospective cohort and meta-analysis. J Neurosurg Spine: 1–11 10.3171/2018.11.SPINE181095 30835707

[17] Korn A, Halevi D, Lidar Z, Biron T, Ekstein P, Constantini S (2015) Intraoperative neurophysiological monitoring during resection of intradural extramedullary spinal cord tumors: experience with 100 cases. Acta Neurochir 157(5):819–830. 10.1007/s00701-014-2307-2 10.1007/s00701-014-2307-2 25514869

[18] Louis DN, Perry A, Reifenberger G, von Deimling A, Figarella-Branger D, Cavenee WK, Ohgaki H, Wiestler OD, Kleihues P, Ellison DW (2016). The 2016 World Health Organization classification of tumors of the central nervous system. A summary Acta Neuropathol.

[19] Mahoney FI, Barthel DW (1965). Functional evaluation. The Barthel Index. Md State Med J.

[20] McCormick PC, Torres R, Post KD, Stein BM (1990). Intramedullary ependymoma of the spinal cord. J Neurosurg.

[21] Odom GL, Finney W, Woodhall B (1958). Cervical disk lesions. J Am Med Assoc.

[22] Ottenhausen M, Ntoulias G, Bodhinayake I, Ruppert F-H, Schreiber S, Förschler A, Boockvar JA, Jödicke A (2019). Intradural spinal tumors in adults-update on management and outcome. Neurosurg Rev.

[23] Rajshekhar V, Velayutham P, Joseph M, Babu KS (2011). Factors predicting the feasibility of monitoring lower-limb muscle motor evoked potentials in patients undergoing excision of spinal cord tumors. J Neurosurg Spine.

[24] Rijs K, Klimek M, Scheltens-de Boer M, Biesheuvel K, Harhangi BS (2019). Intraoperative neuromonitoring in patients with intramedullary spinal cord tumor. A systematic review, meta-analysis, and case series. World Neurosurg.

[25] Sala F, Palandri G, Basso E, Lanteri P, Deletis V, Faccioli F, Bricolo A (2006). Motor evoked potential monitoring improves outcome after surgery for intramedullary spinal cord tumors. A historical control study. Neurosurgery.

[26] Shim HK, Lee JM, Kim DH, Nam KH, Choi BK, Han IH (2021). Successful motor evoked potential monitoring in cervical myelopathy. Related factors and the effect of increased stimulation intensity. J Korean Neurosurg Soc.

[27] Tobimatsu S, Sun SJ, Fukui R, Kato M (1998). Effects of sex, height and age on motor evoked potentials with magnetic stimulation. J Neurol.

[28] Ushirozako H, Yoshida G, Imagama S, Kobayashi K, Ando K, Ando M, Kawabata S, Yamada K, Kanchiku T, Fujiwara Y, Taniguchi S, Iwasaki H, Shigematsu H, Tadokoro N, Takahashi M, Wada K, Yamamoto N, Funaba M, Yasuda A, Hashimoto J, Morito S, Takatani T, Tani T, Matsuyama Y (2021) Efficacy of transcranial motor evoked potential monitoring during intra- and extramedullary spinal cord tumor surgery. A prospective multicenter study of the Monitoring Committee of the Japanese Society for Spine Surgery and Related Research. Global Spine J: 21925682211011443 10.1177/21925682211011443 PMC10189340 34011196

[29] van der Wal EC, Klimek M, Rijs K, Scheltens-de Boer M, Biesheuvel K, Harhangi BS (2021) Intraoperative neuromonitoring in patients with intradural extramedullary spinal cord tumor: a single-center case series. World Neurosurg 147:e516–e523. 10.1016/j.wneu.2020.12.099 10.1016/j.wneu.2020.12.099 33383201

[30] von Elm E, Altman DG, Egger M, Pocock SJ, Gøtzsche PC, Vandenbroucke JP (2014). The Strengthening the Reporting of Observational Studies in Epidemiology (STROBE) statement. Guidelines for reporting observational studies. Int J Surg.

